# Advanced Wound Therapies in the Management of Severe Military Lower Limb Trauma: A New Perspective

**Published:** 2009-07-21

**Authors:** Lt Col S. L. A. Jeffery

**Affiliations:** The Royal Centre for Defence Medicine, Selly Oak Hospital, Birmingham B29 6JD, United Kingdom

## Abstract

**Objective:** The purpose of this article is to describe the treatment of injuries resulting from land mine explosions using a holistic approach that includes gauze-based negative pressure wound therapy (NPWT) and encompasses wound bed preparation, exudate management, and infection control. **Method:** In the treatment of 3 traumatic injuries, each requiring limb amputation, we describe the application of NPWT using the Chariker-Jeter system, which uses a single layer of saline-moistened antimicrobial gauze laid directly onto the wound bed. A silicone drain is placed on the gauze and then more gauze is placed over the drain to fill the wound. This is then covered with a clear semipermeable film, cut so that there is a 2- to 3-cm border around the wound allowing it to be sealed onto healthy skin. **Results:** In each of the cases described, we were able to achieve wound closure prior to successful skin grafting, and the patients have recovered well despite the severity of their injuries. **Conclusion:** We discuss the potential advantages of the Chariker-Jeter system over polyurethane foam as a method of delivering NPWT in highly extensive and irregular-shaped wounds created by land mine explosions while stressing the importance of thorough and effective wound bed preparation.

## INTRODUCTION: HOLISTIC APPROACH TO LARGE, COMPLEX, EXUDATING WOUNDS

Since British forces have been engaged in recent conflicts in Iraq and Afghanistan, the University Hospital Birmingham Foundation Trust and the Royal Centre for Defence Medicine in Birmingham, through which all injured UK troops are transferred for treatment, have been frequently faced with challenging trauma wounds, caused by improvised incendiary devices, land mine explosions, and gunshot wounds. Improvements in body armor have resulted in fewer trunk injuries and therefore relatively more limb injuries, particularly of the lower limbs. These injuries are associated with extensive soft tissue stripping and contamination, and high levels of exudate, and are particularly prone to infection both by bacteria and fungi.

Because of the extent of the trauma involved, our primary strategy was to stabilize the patient and salvage as much of the limb as possible without exposing him or her to an unnecessarily high risk of infection. We have found that a holistic approach that includes negative pressure wound therapy (NPWT) and encompasses wound bed preparation, exudate management, and infection control is an effective way to strive for eventual wound closure and the improvement of the patient's overall condition.

In recent years, NPWT has become an accepted option for managing and treating trauma cases.^1^ It is also beginning to be appreciated more widely both in open fractures of the lower extremity and in both high- and low-energy trauma wounds because of its ability to handle high volumes of exudate and provide a closed wound environment.

With NPWT, subatmospheric pressure is applied to the wound bed through a wound-filler material, which is covered with an airtight seal, and exudate is drawn off into a collection device. Until recently, the only filler available was an open-cell polyurethane (PU) foam, supplied as part of the V.A.C system (KCI, San Antonio, Tex).

An alternative wound filler (nonadherent gauze) is now commercially available in several alternative NPWT devices, including VISTA (Smith & Nephew, Hull, United Kingdom), based on the method now known as the Chariker-Jeter system.^2^ This method uses a single layer of saline-moistened antimicrobial gauze that is impregnated with polyhexamethylene biguanide and laid directly onto the wound bed. A silicone drain is placed on the gauze and then more gauze is placed over the drain to fill the wound. This is then covered with a clear semipermeable film, cut so that there is a 2- to 3-cm border around the wound allowing it to be sealed onto healthy skin.

We have found NPWT with the VISTA device (and a larger version, the EZCARE pump) to be invaluable in managing trauma wounds and featured 3 cases to illustrate how it can be used, within the context of wound bed preparation, to achieve wound closure. In these wounds, NPWT must be used in combination with comprehensive surgical assessment, exploration, and meticulous debridement. Our objectives were to stabilize soft tissue, salvage compromised tissue, reduce edema, infection, wound size, the number and frequency of dressing changes, and finally the complexity of the wound itself to facilitate further reconstructive surgery.

A comparative investigation of the long-term overall cost-effectiveness of this treatment has not yet been undertaken, but the author feels that this could be a very valuable study for the future.

## CASE 1: TRAUMATIC INJURY TO BOTH LEGS, SACRUM, AND BUTTOCKS

**Figure 1:**
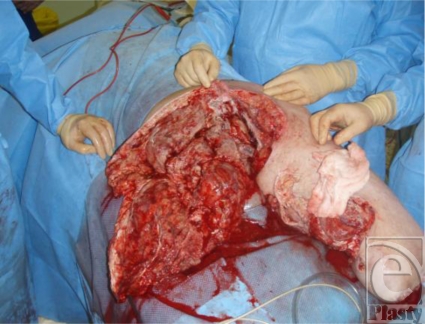
Land mine injury.

A 19-year-old soldier was injured when he stood on a land mine in Afghanistan. He suffered extensive injuries to both his legs and his buttocks. He also suffered abdominal and upper limb injuries (Fig 1). His injury severity score was 25 and his new injury severity score was 34. Microbiological investigation revealed the presence of methicillin-resistant *Staphylococcus aureus*, *Acinetobacter* species, and the *Aspergillus* fungus within the patient's leg wounds.

**Figure 2:**
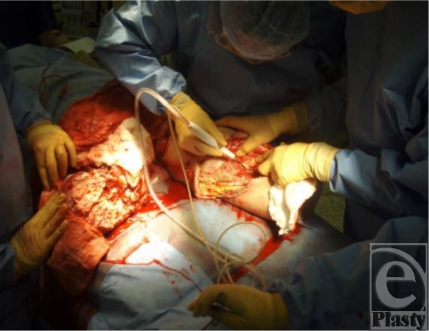
Debridement using the VERSAJET hydrosurgey system.

To begin with, a colostomy was fashioned and extensive debridement of this patient's wounds was carried out with the VERSAJET hydrosurgery system (Smith & Nephew). Meticulous debridement is essential prior to attempted wound closure to promote wound healing and minimize the risk of infection (Fig 2).

Topical NPWT was applied with the VISTA system, as EZCARE was not available then in the United Kingdom. NPWT was applied for 10 days to prepare the wounds for skin grafting.

Before applying NPWT, the wound and surrounding skin were carefully dried: It is essential to do this thoroughly to ensure that a good seal can be maintained when vacuum pressure is applied. We also used an alcohol preparation, such as chlorhexidine in alcohol, to degrease the skin. This was applied around the wound and allowed to dry.

We then created a seal. This is made easier if an adhesive solution is then applied to the skin adjacent to the wound, particularly in areas that are prone to loss of complete seal such as junctional areas (eg, the groin in a high amputation). Whitehead's varnish offers the most adhesion, but tincture of benzoin (Friar's balsam) is also effective. The small sachets of adhesive that are supplied by the manufacturers are sufficient only for small wounds.

Our next procedure was to lay the gauze in the wound. In heavily exudating wounds, we apply gauze while it is still dry rather than wetting it beforehand. It is important to lightly pack the gauze into any crevices so that all parts of the wound are in contact with it.

A drain is then placed onto the gauze, with further gauze packed on top of the drain. Film could then be laid over the entire area to complete the seal.

With this system, negative pressure can frequently be applied at -80 mm Hg. However, for heavily exudating wounds such as we are describing, our practice is to connect the drain and apply suction (on “maximum” setting) before attempting to seal the film. Increasing the pressure temporarily provides a local tamponade effect, removes any exudate that may drip out and ruin the seal, and also helps identify any possible leaks before adjusting the pressure to between -80 and -100 mm Hg. Getting a good seal is critical to the success of the procedure so that theatre staff should take their time and make sure that every part of the skin in contact with the film is absolutely dry. Manufacturers supply “ostomy strip paste,” which can be useful to help seal around the drain, but care should be taken to ensure that none of the holes in the drain are near the paste because there is a risk that the paste will be sucked up into the drain, causing a blockage and therefore a failure of the dressing system.

It is not unusual for some of our large wounds to drain 4.5 L of fluid every 24 hours, and such fluid loss needs to be considered when calculating overall fluid requirements. On occasions, we have used 2 VISTA pumps if there is an exceptionally large amount of exudate to clear. In such cases, we now prefer to use the bigger EZCARE pump, which copes with a higher flow rate.

After a period of 10 days, the wounds were ready for skin grafting. NPWT was applied as the dressing, this time with a layer of Telfa between the skin graft and the gauze to prevent accidental removal of the skin graft at dressing changes.

**Figure 3:**
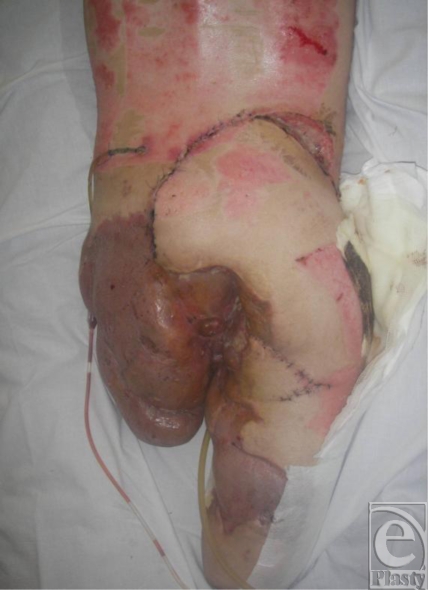
Coverage using a rotation flap.

**Figure 4:**
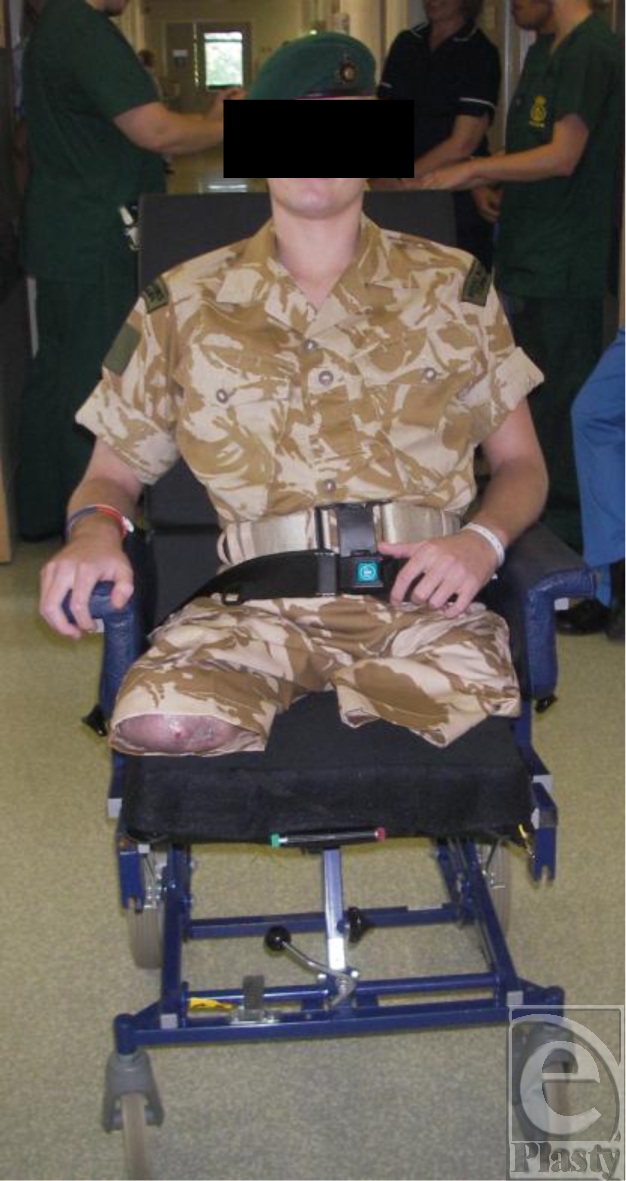
Soldier postsurgery.

Coverage of the remaining sacrum was later achieved with a rotation flap (Fig 3). In spite of his injuries, the soldier's condition continues to improve (Fig 4).

## CASE 2: TRAUMATIC INJURY TO RIGHT LEG

**Figure 5:**
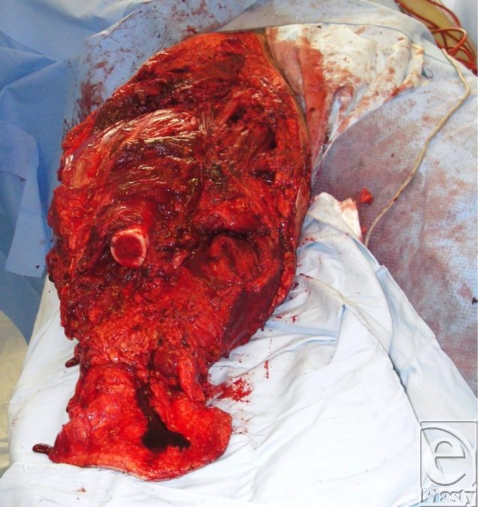
Pressure plate improvised explosive device injury.

**Figure 6:**
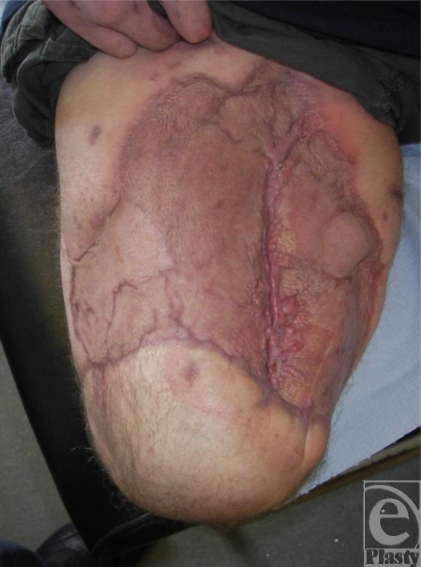
Site of wound after debridement, application of negative pressure wound therapy, and skin grafting.

This soldier was injured when he stood on a pressure plate improvised explosive device in Afghanistan (Fig 5). On arrival in Birmingham, he required emergency above-the-knee amputation of his right leg. Radical debridement left a healthy wound bed, but it was a difficult wound to dress. We did not feel that conventional dressings would have been suitable for this patient. The use of a gauze-based NPWT system (in this case, VISTA) allowed for optimal wound healing, exudate management, and a secure dressing that was not able to slip off. Wound healing was completed with the use of skin grafts, and the soldier now walks with a prosthesis (Fig 6).

## CASE 3: AMPUTATION OF 3 LIMBS

**Figure 7:**
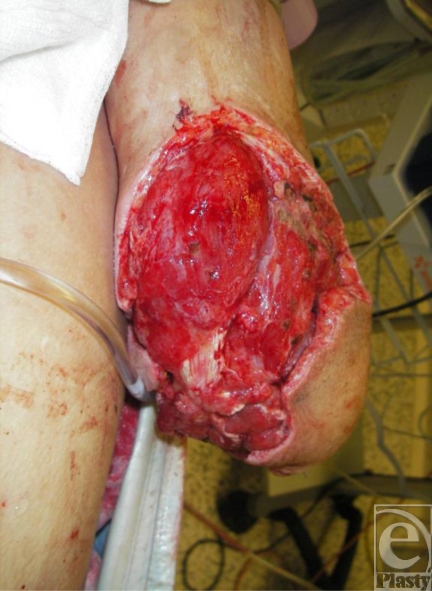
Amputated limb postdebridement.

**Figure 8:**
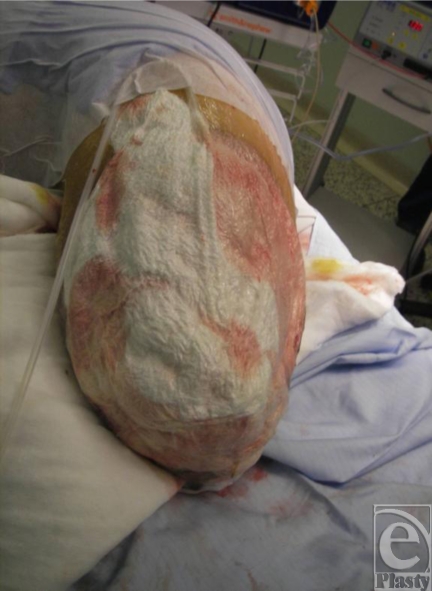
Treatment with gauze-based negative pressure wound therapy over wound area.

A 26-year-old soldier suffered injuries to all 4 of his limbs. He required bilateral above-the-knee amputations and a left above-the-elbow amputation (Fig 7). He also developed a fungal wound infection, requiring multiple debridements, NPWT using gauze provided optimal dressings and exudate management (Fig 8).

**Figure 9:**
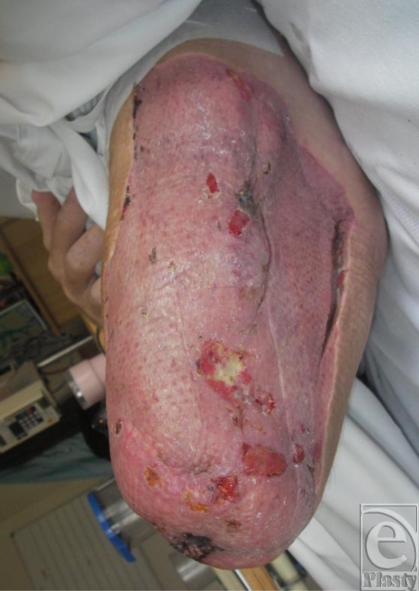
Amputation stump post–skin grafting, following 10 days' negative pressure wound therapy.

Again, after 10 days' treatment with NPWT, skin grafting was performed with gauze-based NPWT (Fig 9). Telfa was used as an antishear layer in the dressing.

## DISCUSSION

Impregnated gauze has recently been “rediscovered” as a filler material for NPWT.^3^–^5^ As the vacuum-assisted closure (VAC) system was the first commercially available negative pressure system, most surgeons will have gained their experience of this therapy using foam and may question whether gauze can produce comparable results. In our experience, the gauze system delivers NPWT effectively and can be considered a reasonable alternative to foam. We also mention in the following text some areas where it can provide specific advantages in the types of wounds described in this article.

Malmsjö et al^6^,^7^ found that in open wounds, gauze is comparable with PU foam in terms of pressure transduction, wound contraction, and stimulation of blood flow at the wound edge. It has also been shown that reduction in wound size progresses at a similar rate, around 15% per week, regardless of the type of wound filler used.^8^

**Figure 10:**
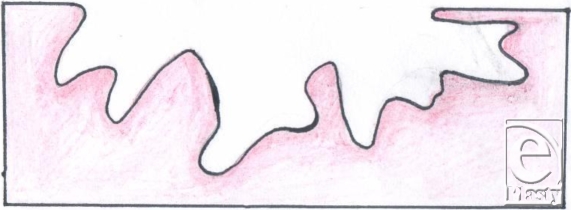
A typical wound shape resulting from an explosive device.

Although foam is widely used with negative pressure systems,^9^ personal experience indicates that it can present a number of problems. First, it can be difficult to cut into shape to fit a wound. Many wounds, especially those resulting from explosive devices, are highly irregular in shape and may extend over a large area and over curved surfaces of the body (Fig 10). While the foam pad has a side that is perpendicular to the base, this is rarely found in actual wounds. As the foam has to be cut into shape, there is a risk of small bits of foam becoming lost in the procedure, either through dropping onto the floor or, worse, into the wound. No incidence of retained gauze has as yet been experienced after NPWT at the Royal Centre for Defence Medicine. Large rolls of gauze are normally used, and if it is inserted into deep structures, a longer tail would be left hanging into the main wound. We also ensure that sequential images are taken at each dressing change to ensure continuity of practice while some key staff member from the surgical or nursing team is retained when there is a change of shift.

Because the bed of a wound is usually contoured whereas the foam pad is completely flat, the flat surface of the foam pad comes into contact only with part of the wound bed. Although the foam pad is meant to set itself to the wound as soon as the vacuum is applied, our opinion, based on our experience, is that this does not always take place with complete effectiveness on irregular-shaped wounds. The filler material always needs to be in contact with the wound surface for suction to be at its most effective.^6^ When this does not take place, this means that parts of the wound bed are not being treated. Furthermore, if the wound base is curved, as often happens with large wounds that extend around the surface of a leg or an arm, the “shape memory” of the foam causes it to lift up out of the wound until it is sealed. It therefore usually requires several members of the theatre team to hold the foam in place and then to apply the covering drape to achieve a complete seal.

**Figure 11:**
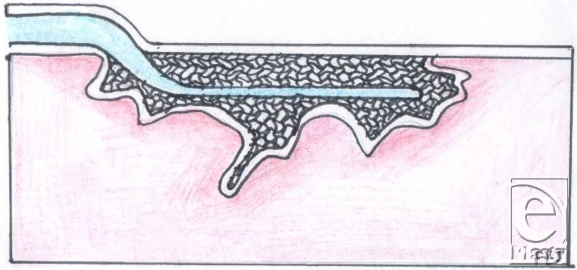
Gauze packed within a typical wound shape.

**Figure 12:**
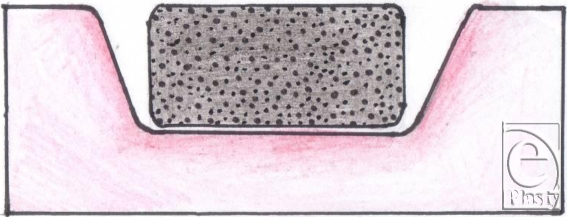
Shape of wound more suitable for sponge.

**Figure 13:**
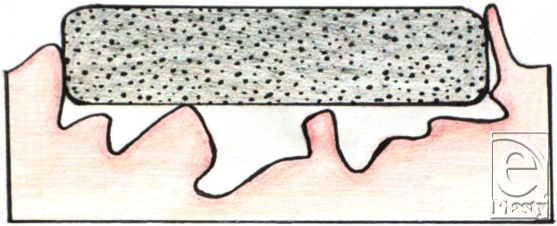
Foam placed in an irregular wound shape.

**Figure 14:**
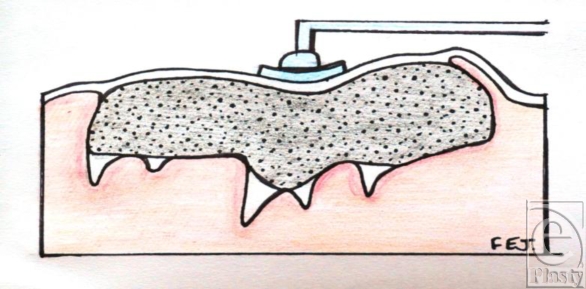
Foam under pressure in an irregular wound shape.

Finally, large wounds require multiple pieces of foam to be “tiled” into the wound and these need to be stapled to each other. For this and other reasons, we have found it easier to work with gauze. It is a very simple procedure to lay gauze into the wound, where it naturally assumes the contours of the wound bed, and to cut it to shape. Furthermore, it can easily be packed into crevices, pockets, or tunnels (Figs 11–14).

The PU foam filler shrinks when vacuum is applied to it, which can be a mixed blessing. Although this is an advantage for wounds healing by secondary intention, it is not desirable in wounds that have, or will need, a skin graft or a flap. It is also a disadvantage in wounds around a joint, where shrinkage causes contraction of the joint, and in some elective releases carried out in burns patients. A surgical release is often carried out in the anticipation that a skin graft will cause shrinkage. The VAC system immediately shrinks the wound, although it would appear that gauze does not suffer so much shrinkage in the lateral direction.

A problem that other users have noted in several reports is the ingrowth of granulation tissue into the cells of PU foam, causing severe pain at dressing changes and disturbance of the reepithelialization process.^10^–^16^

In all, the gauze-based Chariker-Jeter system may have some advantages at dressing changes in reducing pain and causing minimal disturbance to fragile granulation tissue.^17^ There have been reports of patients requiring sedation at dressing changes with the foam-based VAC system,^8^ but we have found patients are able to tolerate removal of the nonadherent gauze dressing used in the VISTA system. There have been no reports of adhesion and consequently no need to debride between applications, therefore helping provide increased comfort and reduced pain overall.

Kerlix gauze is impregnated with polyhexamethylene biguanide that has an antimicrobial action largely residing within the fluid transfer conduit. Until recently, the PU foam in the VAC system did not have antimicrobial properties but this has now been modified to contain micro-bonded metallic silver. The levels released are quoted on the KCI Web site to be in the range of 3 ppm over a 72-hour period.

It would be unwise to rely entirely on antimicrobials incorporated into the fluid transfer component to provide protection for the wound against infection. In at-risk wounds, interventional approaches with antibiotics and/or effective bactericidal antibacterial delivery systems such as nanocrystalline silver may be clinically required prior to, or during NPWT use, to help control infection. In our experience, thorough debridement should always be carried out prior to NPWT to reduce bacterial load and the use of topical antimicrobials should be considered on the basis of an assessment of the pathogens most likely to be present in the wound.

## CONCLUSION

In the management of complex wounds, there are clinical benefits to be gained from a holistic approach involving topical NPWT in combination with wound bed preparation and infection control strategies. We have found the gauze-based VISTA system to be versatile and capable of being applied to a wide variety of wounds that because of their location, extent, and level of exudate would be difficult to manage with conventional dressings. The gauze wound filler used in the VISTA system also has some positive advantages for the surgical team regarding its conformability and ease of application to large and irregular wounds. Finally, there are benefits for the patient in terms of comfort and reduced pain on removal compared with that sometimes reported with PU foam.
